# Comparative clinical and prognostic significance of integrin αvβ3, αvβ5, and αvβ6 in osteosarcoma

**DOI:** 10.3389/fonc.2026.1888679

**Published:** 2026-08-04

**Authors:** Chaoqun You, Fanwei Zeng, Shuai Zhang, Jiuhui Xu, Tingting Ren, Lu Xie, Yuchen Song, Xiaodong Tang, Ran Wei

**Affiliations:** 1 Musculoskeletal Tumor Center, Peking University People’s Hospital, Beijing, China; 2 Beijing Key Laboratory of Musculoskeletal Tumor, Beijing, China

**Keywords:** integrin αvβ3, integrin αvβ5, integrin αvβ6, osteosarcoma, prognosis

## Abstract

**Background:**

Integrins are involved in extracellular matrix interactions and tumor progression, but the comparative clinical relevance of αvβ3, αvβ5, and αvβ6 in osteosarcoma remains unclear.

**Methods:**

This retrospective cohort included 193 patients with histologically confirmed osteosarcoma and available formalin-fixed, paraffin-embedded tumor specimens, evaluable immunohistochemical staining, and follow-up data. Corresponding hematoxylin and eosin-stained sections were reviewed to identify viable osteosarcoma-containing regions. H-score assessment for αvβ3, αvβ5, and αvβ6 was restricted to morphologically identifiable viable osteosarcoma cells, while non-tumoral staining was excluded. Two observers blinded to clinical data independently evaluated staining intensity and the percentage of positive tumor cells. Median H-scores were used for Kaplan–Meier curve visualization, whereas H-scores were modeled continuously in Cox regression analyses. Event-free survival (EFS) and metastasis-free survival (MFS) were analyzed using Kaplan–Meier and Cox models adjusted for age, sex, and tumor necrosis rate. MFS analyses included 183 patients without metastatic disease at baseline. Exploratory combined-expression analyses were also performed.

**Results:**

All three integrins showed heterogeneous expression in osteosarcoma tissues. αvβ3 expression was significantly higher in patients who developed metastatic events during follow-up, whereas αvβ5 and αvβ6 were not associated with metastatic events. High αvβ3 expression was associated with poorer EFS (p = 0.003) and MFS (p = 0.001). In multivariable models, continuous αvβ3 H-score remained associated with poorer EFS (HR, 1.011; 95% CI, 1.005–1.017; p < 0.001) and MFS (HR, 1.012; 95% CI, 1.006–1.019; p < 0.001). αvβ5 and αvβ6 were not significantly associated with survival, and combined-expression analyses did not provide clear additional prognostic discrimination.

**Conclusions:**

Among the three αv-containing integrins examined, αvβ3 showed the most consistent association with metastatic events during follow-up and poorer EFS and MFS. Its long-term prognostic relevance and clinical applicability require validation in larger independent cohorts with longer follow-up.

## 1 Introduction

Osteosarcoma is the most common primary malignant bone tumor in children, adolescents, and young adults. Despite advances in multi-agent chemotherapy and standardized surgical management, it remains a highly aggressive disease characterized by early metastatic spread and substantial biological heterogeneity (1–3). Although overall survival has improved over recent decades, the prognosis remains poor for patients who develop metastasis, recurrence, or resistance to treatment. Lung metastasis is particularly frequent and continues to be a major cause of osteosarcoma-related death (1, 2, 4). These clinical challenges highlight the need for reliable biomarkers that can reflect tumor aggressiveness and improve risk stratification in osteosarcoma (5, 6).

Integrins are transmembrane adhesion receptors that mediate interactions between cells and the extracellular matrix while also regulating a wide range of intracellular signaling pathways (7). Through these functions, they influence key malignant phenotypes, including tumor cell survival, migration, invasion, angiogenesis, and adaptation to the tumor microenvironment (8, 9). Among the integrin family, αv-containing integrins have attracted particular attention in cancer research because of their close involvement in tumor progression and metastasis (10). Different αv integrins interact with distinct extracellular ligands and may activate overlapping, but not identical, downstream signaling networks. As a result, individual members of this family may have different biological functions and clinical significance even within the same tumor type (11).

Among αv-containing integrins, αvβ3, αvβ5, and αvβ6 are of particular interest because they have been implicated in extracellular matrix remodeling, invasive behavior, angiogenesis, and metastatic dissemination in multiple malignancies (12, 13). However, their roles appear to vary according to tumor type and biological context (14). Previous osteosarcoma studies have implicated αvβ3 and αvβ5 in metastatic progression and suggested that targeting these integrins may suppress pulmonary metastasis in preclinical models. In contrast, the clinical significance of αvβ6 in osteosarcoma remains less well characterized; therefore, these three αv-containing integrins were evaluated as a focused comparative panel in the present study (12). In osteosarcoma, previous studies have suggested that integrin-related pathways contribute to tumor progression, cell adhesion, migration, and interaction with the bone microenvironment (15–17). Nevertheless, the comparative clinical value of αvβ3, αvβ5, and αvβ6 in osteosarcoma remains unclear. It is not yet known whether these three markers provide similar prognostic information, whether one marker is more informative than the others, or whether combined assessment offers additional value beyond single-marker analysis.

In the present study, we performed immunohistochemical evaluation of αvβ3, αvβ5, and αvβ6 in a retrospective osteosarcoma cohort with follow-up data. We compared their expression patterns, analyzed their associations with major clinicopathological events, and assessed their prognostic value for event-free survival and metastasis-free survival. We also explored the relationships among these three markers and further examined whether combined stratification based on the three-integrin panel could improve prognostic discrimination. Our aim was to clarify the relative clinical significance of αvβ3, αvβ5, and αvβ6 in osteosarcoma and to identify the most informative marker within this panel.

## 2 Materials and methods

### 2.1 Patients and tissue specimens

This retrospective study included 193 patients with osteosarcoma who were treated at the Department of Bone Tumor at Peking University People’s Hospital between 2023 and 2024. Formalin-fixed, paraffin-embedded (FFPE) tumor tissue specimens were collected for immunohistochemical analysis. Clinicopathological data were obtained from the medical records, including age, sex, tumor site, metastasis status, recurrence status, tumor necrosis rate, and other available variables.

**Figure 1:**
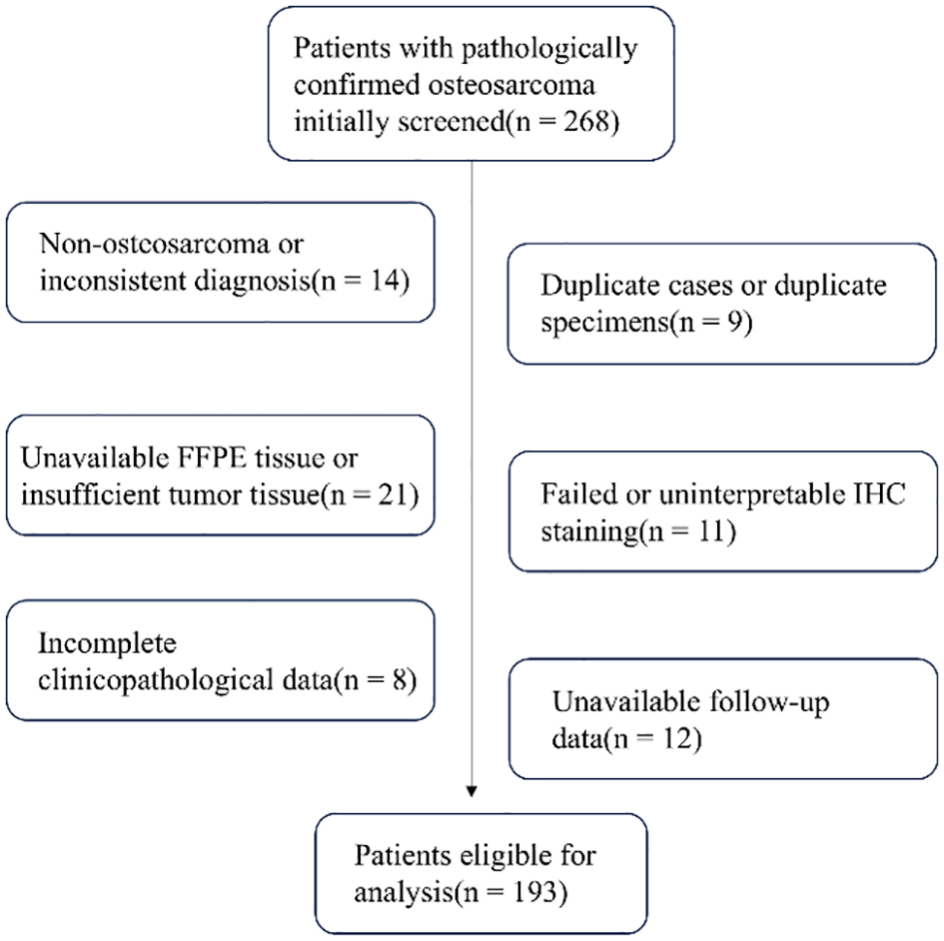
Flowchart of patient selection in this retrospective osteosarcoma cohort.

This was a retrospective consecutive cohort study. All potentially eligible cases identified during the predefined study period were consecutively screened according to the prespecified inclusion and exclusion criteria. As shown in **Figure 1**, 268 cases were initially screened, and 193 patients with evaluable immunohistochemical results and complete clinicopathological and follow-up data were ultimately included in the final analysis.

During follow-up, 101 EFS events were observed among the 193 included patients. For MFS analysis, 10 patients with metastatic disease at baseline were excluded, leaving 183 patients at risk, among whom 82 developed metastatic events during follow-up.

The inclusion criteria were as follows: (1) histopathologically confirmed osteosarcoma; (2) availability of FFPE tumor tissue specimens for immunohistochemical analysis; (3) successful immunohistochemical staining and H-score evaluation for integrin αvβ3, αvβ5, and αvβ6; and (4) available clinicopathological and follow-up data for subsequent analysis. The exclusion criteria were as follows: (1) non-osteosarcoma or uncertain pathological diagnosis; (2) duplicate cases or repeated specimens from the same patient; (3) insufficient tumor tissue, poor tissue preservation, or unavailable FFPE specimens for immunohistochemical staining; (4) failed staining or uninterpretable immunohistochemical results; and (5) incomplete clinicopathological data or unavailable follow-up information.

### 2.2 Immunohistochemistry

FFPE tissue sections with a thickness of 4 μm were prepared for immunohistochemical staining. After deparaffinization and rehydration, antigen retrieval was performed using Ethylenediaminetetraacetic acid for integrin αvβ3 and sodium citrate for integrins αvβ5 and αvβ6. Endogenous peroxidase activity was blocked with 3% H_2_O_2_ solution for 15 min. The sections were then incubated with primary antibodies against integrin αvβ3, αvβ5, and αvβ6 at 4 °C for overnight.

The primary antibodies used were as follows: αvβ3 (catalog number GTX01084, dilution 1:300), αvβ5 (catalog number bs-1356R, dilution 1:200), and αvβ6 (catalog number bs-5791R, dilution 1:200). After washing, the sections were incubated with the appropriate secondary antibody at room temperature for 1 h. Immunoreactivity was visualized with 3,3’-Diaminobenzidine, and the sections were counterstained with hematoxylin. Negative controls were prepared by replacing the primary antibody with PBS.

### 2.3 Evaluation of immunohistochemical staining

Immunohistochemical staining was evaluated independently by 2 observers who were blinded to the clinical data. The expression levels of αvβ3, αvβ5, and αvβ6 were assessed using the H-score method. Staining intensity was scored as 0, 1, 2, or 3, corresponding to negative, weak, moderate, and strong staining, respectively. The percentage of positively stained tumor cells was recorded for each intensity level.

The H-score was calculated as follows:

H-score = (percentage of cells with intensity 1 × 1) + (percentage of cells with intensity 2 × 2) + (percentage of cells with intensity 3 × 3).

The final H-score ranged from 0 to 300. For survival analysis, patients were divided into low- and high-expression groups according to the median H-score of each marker.

For each case, the corresponding hematoxylin and eosin-stained section was reviewed to identify viable osteosarcoma-containing regions. Within these regions, H-score assessment was restricted to morphologically identifiable viable osteosarcoma cells. Non-tumoral staining in endothelial cells, stromal fibroblasts, inflammatory cells, osteoclast-like giant cells, non-neoplastic osteoid-associated cells, and necrotic areas was not included in the analysis. The same scoring principle was applied to integrin αvβ3, αvβ5, and αvβ6.

### 2.4 Follow-up and survival endpoints

Follow-up data were obtained from medical records, outpatient records, and the institutional follow-up database. The main endpoints of this study were event-free survival (EFS) and metastasis-free survival (MFS). EFS was defined as the time from surgery to the first occurrence of metastasis, recurrence, death, or last follow-up. MFS was defined as the time from surgery to the occurrence of metastasis or last follow-up. Metastatic events occurring during follow-up were treated as outcome events and were therefore not included as covariates in the Cox regression models. Patients without events were censored at the date of the last follow-up. The median follow-up time was 20.4 months (interquartile range, 14.2–29.4 months). During follow-up, 101 EFS events were observed among 193 patients. For MFS analysis, 10 patients with metastatic disease at baseline were excluded, leaving 183 patients at risk, among whom 82 developed metastatic events during follow-up.

### 2.5 Correlation analysis and combined stratification analysis

The correlations among αvβ3, αvβ5, and αvβ6 expression levels were assessed using Spearman correlation analysis. Correlation coefficients were reported as ρ values.

To explore the combined prognostic value of the three integrins, several combined stratification methods were used. Patients were first classified into eight groups according to all possible low/high combinations of αvβ3, αvβ5, and αvβ6 expression. Patients were also classified according to the number of highly expressed integrins as [0–1 high vs. 2–3 high]. In addition, a six-group joint classification model was constructed based on the combined expression patterns of the three markers.

### 2.6 Statistical analysis

All statistical analyses were performed using R software (version 4.5.2). Continuous variables were expressed as median [interquartile range], and categorical variables were expressed as number (percentage).

Differences in H-scores between two groups were compared using an independent-samples t test. Correlations among αvβ3, αvβ5, and αvβ6 were analyzed using Spearman correlation analysis.

Survival curves were generated using the Kaplan-Meier method and compared using the log-rank test. The prognostic value of each variable was assessed by univariable and multivariable Cox proportional hazards regression analyses. The results were presented as hazard ratios (HRs) and 95% confidence intervals (CIs).

An event-based sensitivity analysis was performed using Schoenfeld’s formula for a two-sided log-rank test to estimate the minimum detectable hazard ratio for the primary αvβ3 survival comparisons at 80% power and an alpha level of 0.05.

All statistical tests were two-sided, and P < 0.05 was considered statistically significant.

## 3 Results

### 3.1 Expression of integrin αvβ3, αvβ5, and αvβ6 in osteosarcoma tissues

As shown in **Figure 1**, a total of 268 patients with pathologically confirmed osteosarcoma were initially screened. After excluding cases with non-osteosarcoma or inconsistent diagnoses, duplicate cases or specimens, unavailable or insufficient FFPE tumor tissue, failed or uninterpretable IHC staining, incomplete clinicopathological data, or unavailable follow-up data, 193 osteosarcoma cases were finally included in this study. All included cases had available clinicopathological data, immunohistochemical staining results, and follow-up information. The main available demographic and clinicopathological characteristics and follow-up outcomes of the cohort are summarized in **Table 1**.

**Table 1:** Main available clinical characteristics and follow-up outcomes of the osteosarcoma cohort.

Characteristic	Overall cohort (n = 193)
Age, years	15.0 (10.0–21.0)
Male sex	121 (62.7)
Tumor necrosis rate, %	81.5 (62.3–90.6)
Follow-up duration, months	20.4 (14.2–29.4)
EFS events	101 (52.3)
Metastatic events during follow-up	92 (47.7)

Data are presented as median (interquartile range) or n (%).

**Figure 2:**
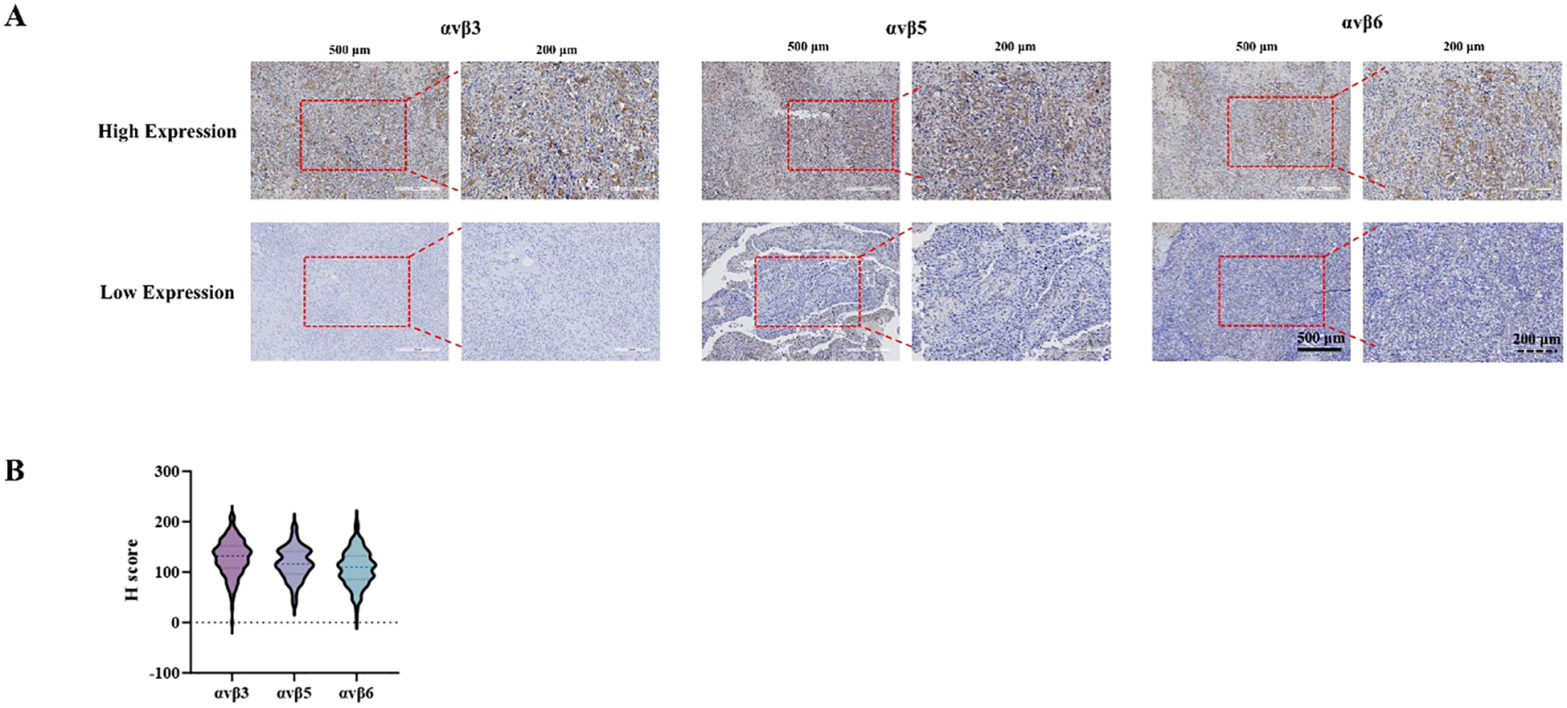
Expression patterns of integrin αvβ3, αvβ5, and αvβ6 in osteosarcoma tissues. **(A)** Representative immunohistochemical staining images of low- and high-expression cases for integrin αvβ3, αvβ5, and αvβ6, showing inter-case heterogeneity in staining intensity and distribution. Images at two magnifications are presented for each marker. Dashed and solid scale bars indicate 200 μm and 500 μm, respectively. **(B)** Violin plots showing the distribution of H-scores for αvβ3, αvβ5, and αvβ6 in the whole cohort.

Immunohistochemical staining demonstrated that integrin αvβ3, αvβ5, and αvβ6 were all expressed in osteosarcoma tissues, although their expression levels varied substantially among cases. Some tumors showed weak staining, whereas others exhibited much stronger staining, and the extent of staining also differed across specimens (**Figure 2**). H-score evaluation further confirmed this heterogeneity, showing a relatively broad distribution of expression levels for all three integrins in the cohort. These findings suggest that αvβ3, αvβ5, and αvβ6 are not uniformly expressed in osteosarcoma and may therefore have distinct biological and clinical roles.

### 3.2 Association of integrin expression with clinicopathological characteristics

**Figure 3:**
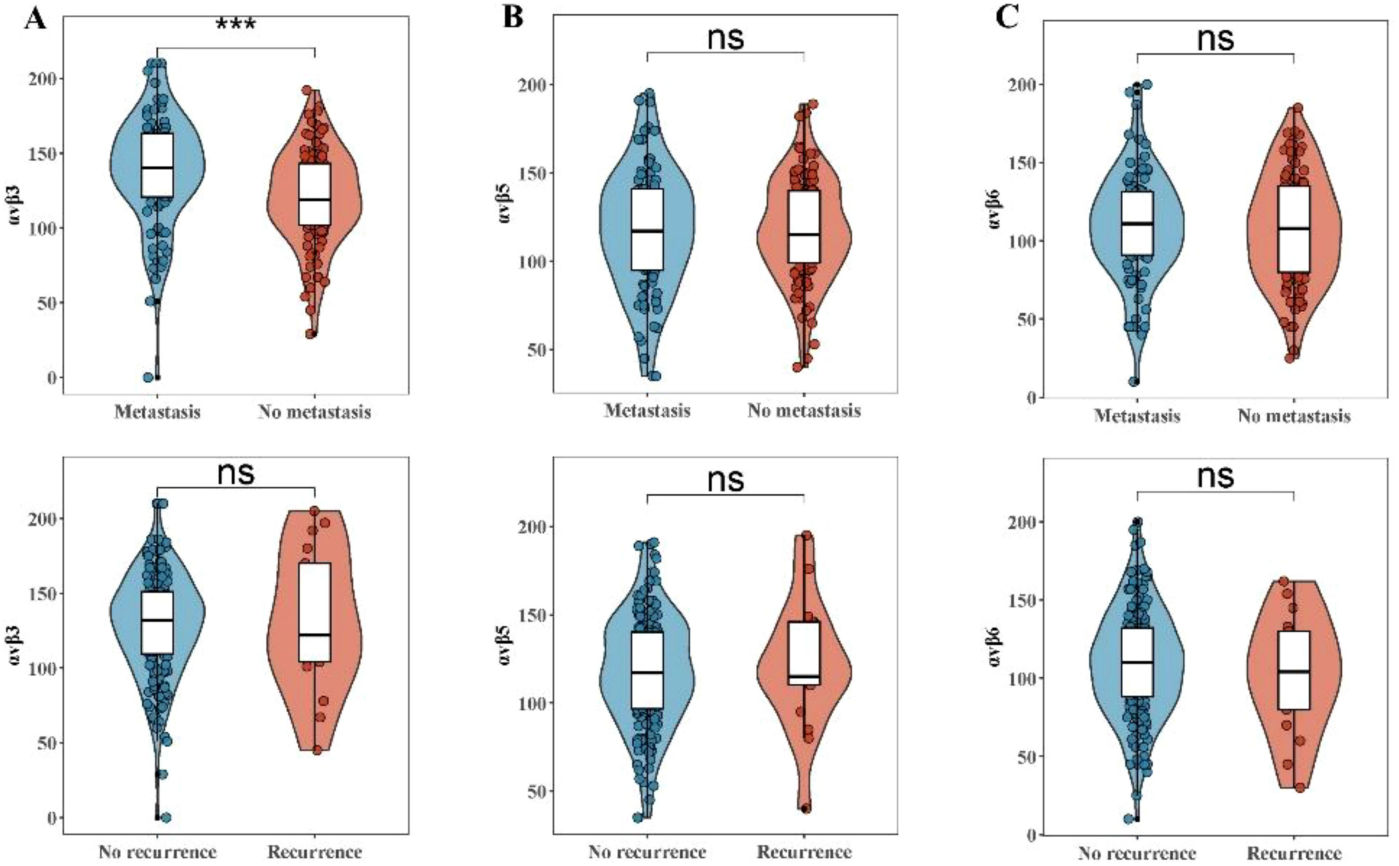
Associations between integrin expression and clinicopathological events in osteosarcoma. **(A–C)** Comparison of αvβ3, αvβ5, and αvβ6 H-scores between patients with and without metastatic events during follow-up. **(D–F)** Comparison of αvβ3, αvβ5, and αvβ6 H-scores between patients with and without recurrence during follow-up. Violin plots with embedded boxplots and individual data points are shown. “***” indicates p < 0.001, and “ns” indicates not significant.

We next analyzed the relationship between integrin expression and major clinical events. Among the three markers, αvβ3 showed the clearest association with clinical aggressiveness. Its expression level was significantly higher in patients with metastasis than in those without metastasis (**Figure 3A**). In contrast, αvβ5 and αvβ6 were not significantly associated with metastatic events during follow-up (**Figures 3B, C**). For recurrence, none of the three integrins showed a significant difference between the recurrence and non-recurrence groups (**Figures 3D–F**).

This result suggests that αvβ3 is more closely related to metastatic behavior than αvβ5 or αvβ6. This point is important because metastasis is one of the main causes of poor outcome in osteosarcoma. The finding also agrees with previous biological knowledge. αvβ3 has been linked to tumor cell migration, invasion, and interaction with the extracellular matrix in several cancers. Although our study was not designed to test mechanism, the present data support a closer link between αvβ3 and an aggressive osteosarcoma phenotype.

We also examined the relationships among the three integrins themselves. Spearman analysis showed a weak positive correlation between αvβ3 and αvβ5 (ρ = 0.20, p = 0.0061). However, αvβ3 was not significantly correlated with αvβ6 (ρ = 0.085, p = 0.24), and αvβ5 was not significantly correlated with αvβ6 (ρ = 0.088, p = 0.22) (**Supplementary Figure 1**). These results suggest that the three markers are not strongly collinear. They may reflect partly different biological features in osteosarcoma.

### 3.3 Prognostic value of αvβ3, αvβ5, and αvβ6 in osteosarcoma

**Figure 4:**
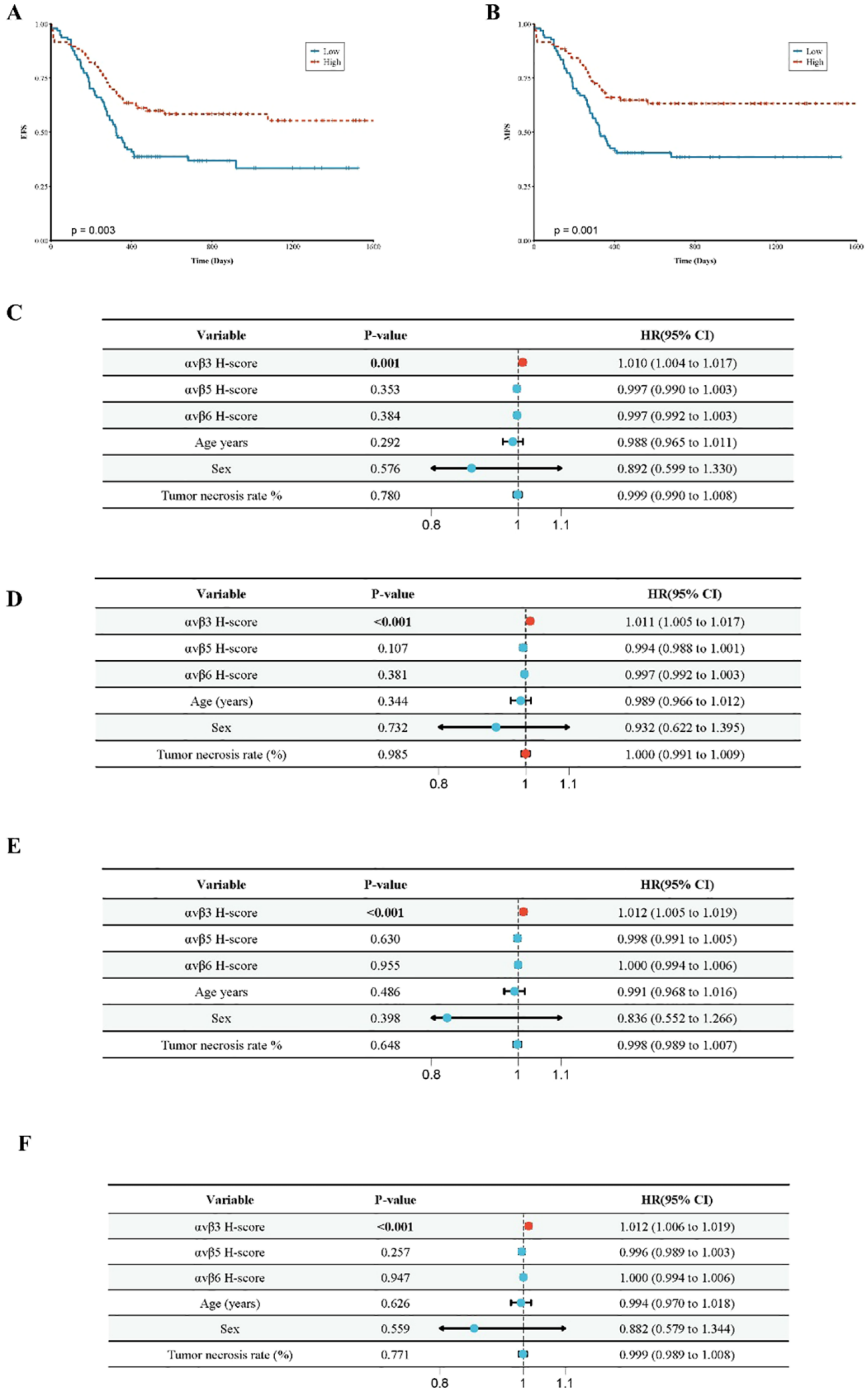
Association of integrin αvβ3 expression with EFS and MFS in the osteosarcoma cohort. **(A, B)** Kaplan–Meier curves for EFS and MFS according to low and high αvβ3 expression groups defined by the median H-score. **(C)** Univariable Cox regression analysis for EFS. **(D)** Multivariable Cox regression analysis for EFS adjusted for age, sex, and tumor necrosis rate. **(E)** Univariable Cox regression analysis for MFS. **(F)** Multivariable Cox regression analysis for MFS adjusted for age, sex, and tumor necrosis rate.

We then assessed the prognostic value of these three integrins. Patients were divided into low- and high-expression groups based on the median H-score. Kaplan–Meier analysis showed that high αvβ3 expression was associated with significantly worse EFS and MFS. The p values were 0.003 for EFS and 0.001 for MFS (**Figures 4A, B**). Based on the available event counts, the minimum detectable hazard ratios at 80% power were 1.75 for EFS and 1.86 for MFS (**Table 2**).

**Table 2:** Event-based sensitivity analysis for the primary αvβ3 survival comparisons.

Outcome	Patients analyzed	Low/high αvβ3 groups	Events	Minimum detectable HR at 80% power
EFS	193	96 / 97	101	1.75
MFS	183	88 / 95	82	1.86

By contrast, αvβ5 did not significantly stratify survival. The p values were 0.743 for EFS and 0.960 for MFS. αvβ6 also showed no significant association with survival, with p values of 0.881 for EFS and 0.712 for MFS (**Supplementary Figure 2**). Taken together, these survival curves show that αvβ3 had the most stable prognostic signal among the three markers.

We further tested this finding by Cox regression analysis. In univariable analysis, αvβ3 H-score was significantly associated with poorer EFS, with an HR of 1.010 (95% CI, 1.004–1.017; p = 0.001). For MFS, the HR was 1.012 (95% CI, 1.005–1.019; p < 0.001). αvβ5 and αvβ6 were not significant in either model (**Figure 4C, E**).

In multivariable analysis, the result remained stable. After adjustment for age, sex, and tumor necrosis rate, αvβ3 was still independently associated with worse EFS and MFS. The HR for EFS was 1.011 (95% CI, 1.005–1.017; p < 0.001). The HR for MFS was 1.012 (95% CI, 1.006–1.019; p < 0.001). Again, αvβ5 and αvβ6 were not significant (**Figures 4D, F**). These findings were consistent across different analyses. The Kaplan–Meier curves and Cox models both pointed to the same conclusion. Among αvβ3, αvβ5, and αvβ6, αvβ3 showed the most consistent association with poorer EFS and MFS in the present cohort. These findings suggest that αvβ3 may be a candidate marker associated with adverse clinical outcomes in osteosarcoma.

### 3.4 Exploratory combined stratification analysis of the three-integrin panel

Because αvβ3, αvβ5, and αvβ6 were evaluated together, we also explored whether their combined expression patterns could improve prognostic stratification. The overall results were limited. In the eight-group model based on all low/high combinations, the association was not significant for EFS (p = 0.112) and only marginally significant for MFS (p = 0.049) (**Supplementary Figures 3A–F**).We also tested simpler grouping methods. When patients were divided by the number of highly expressed integrins, the results were still weak. The p values were 0.167 for EFS and 0.061 for MFS (**Supplementary Figures 3C, D**). The six-group joint classification gave similar findings, with p = 0.082 for EFS and p = 0.054 for MFS (**Supplementary Figures 3E, F**).

These exploratory analyses suggest that combining the three markers did not lead to a clear improvement in prognostic performance. The main prognostic information still came from αvβ3 alone. This may be because αvβ5 and αvβ6 contributed limited additional information in this cohort. Therefore, although the three-integrin panel is of biological interest, the current data support αvβ3 as the main clinically informative marker.

Overall, the present study showed that αvβ3, αvβ5, and αvβ6 were all expressed in osteosarcoma tissues, but their clinical significance was different. Among the three markers, αvβ3 showed the strongest link with metastasis and the clearest association with poor survival. It also remained significant after multivariable adjustment. In contrast, αvβ5 and αvβ6 showed limited clinicopathological and prognostic value. These findings suggest that αvβ3 is the most clinically relevant integrin among the three examined markers in osteosarcoma.

## 4 Discussion

In this study, we comparatively evaluated the expression patterns and clinical significance of integrin αvβ3, αvβ5, and αvβ6 in osteosarcoma. Although all three markers were expressed in tumor tissues, their clinical relevance was not equivalent. Among them, αvβ3 showed the clearest association with metastatic status and the most consistent prognostic value for both event-free survival and metastasis-free survival. In contrast, αvβ5 and αvβ6 showed limited associations with clinicopathological events and survival outcomes. Overall, these findings suggest that αvβ3 may be the most clinically informative integrin among the three examined markers in osteosarcoma.

A key finding of the present study is that αvβ3 expression was significantly higher in patients with metastasis, whereas αvβ5 and αvβ6 were not significantly associated with metastatic status. This observation is clinically meaningful because metastasis remains the major determinant of adverse outcome in osteosarcoma (3). Integrin αvβ3 has been implicated in tumor cell adhesion, migration, invasion, and extracellular matrix interaction in several malignancies, and previous studies have also suggested a role for αv-related integrin signaling in osteosarcoma progression (18, 19). In this context, our results support the view that αvβ3 is more closely linked to an aggressive osteosarcoma phenotype than αvβ5 or αvβ6. Although the current study was not designed to investigate molecular mechanisms, the clinical association observed here is consistent with the known pro-invasive and pro-metastatic functions of αvβ3.

The survival analyses further strengthened this conclusion. Kaplan–Meier analysis showed that high αvβ3 expression was significantly associated with worse EFS and MFS, whereas αvβ5 and αvβ6 did not significantly stratify survival. More importantly, αvβ3 remained significant in both univariable and multivariable Cox regression models after adjustment for age, sex, and tumor necrosis rate. The association between αvβ3 expression and poorer EFS/MFS remained statistically significant after adjustment for the available clinical covariates, including age, sex, and tumor necrosis rate. This finding further supports the potential clinical relevance of αvβ3 expression in osteosarcoma. By comparison, αvβ5 and αvβ6 appear to provide less prognostic information in the present dataset.

Another noteworthy finding is that the correlations among αvβ3, αvβ5, and αvβ6 were generally weak. Only αvβ3 and αvβ5 showed a weak positive correlation, while neither of them was significantly correlated with αvβ6. These data suggest that the three markers are not strongly collinear and may reflect partly distinct biological features in osteosarcoma. However, despite this relative independence, combined stratification based on the three-integrin panel did not substantially improve prognostic discrimination. Whether using the eight-group model, the number-of-high-integrins model, or the six-group joint classification model, the results were either non-significant or only marginally significant. Therefore, the additional prognostic contribution of αvβ5 and αvβ6 appeared limited, and the main clinically relevant signal in this cohort was still driven by αvβ3.

These findings may help clarify the comparative value of different αv-containing integrins in osteosarcoma. Although αvβ3, αvβ5, and αvβ6 all belong to the same integrin subfamily, their biological roles may differ according to ligand preference, downstream signaling context, and tumor microenvironment (17, 20). This may partly explain why all three markers were detectable in osteosarcoma tissues, but only αvβ3 showed clear clinicopathological and prognostic relevance. The current results therefore support the importance of evaluating integrin members individually rather than assuming that all αv-containing integrins have similar clinical significance within the same tumor type.

Several limitations of this study should be acknowledged. First, this was a retrospective single-center study, which may be subject to selection bias. Second, although the sample size was adequate to detect the dominant effect of αvβ3, it may still have limited the power of subgroup analyses and combined-marker analyses. Third, the median follow-up duration was relatively limited, which may have reduced the ability to capture late recurrence, metastasis, and survival events. Therefore, although higher αvβ3 expression was associated with poorer EFS and MFS in the present cohort, the current findings should not be interpreted as definitive evidence of long-term prognostic utility. In addition, comprehensive adjustment for all clinically relevant baseline prognostic factors was not feasible in this retrospective dataset, and residual confounding cannot be excluded. Larger multicenter cohorts with longer follow-up and more complete baseline clinical information are required to validate the long-term clinical relevance of αvβ3 in osteosarcoma.

In summary, the present study shows that αvβ3, αvβ5, and αvβ6 are all expressed in osteosarcoma tissues, but their clinical significance is not equivalent. Among them, αvβ3 demonstrated the strongest association with metastasis and the clearest prognostic value for both EFS and MFS. By contrast, αvβ5 and αvβ6 showed limited clinical relevance in this cohort. These findings support αvβ3 as the most promising integrin marker among the three examined candidates for prognostic assessment in osteosarcoma.

## 5 Conclusion

In conclusion, integrin αvβ3, αvβ5, and αvβ6 were all expressed in osteosarcoma tissues, but their clinical significance was not the same. Among the three markers, αvβ3 showed the strongest association with metastatic status and the clearest prognostic value for both event-free survival and metastasis-free survival. In contrast, αvβ5 and αvβ6 showed limited clinical and prognostic relevance in this cohort. Combined analysis of the three-integrin panel provided only modest additional value. These findings identify αvβ3 as the integrin most consistently associated with metastatic events during follow-up and poorer EFS and MFS among the three markers examined. Its potential long-term prognostic value and clinical applicability require validation in larger independent cohorts with longer follow-up.

## Data Availability

The de-identified data supporting the findings of this study are available from the corresponding author upon reasonable request, subject to institutional and ethical approval.
