# Supplementary material for: Comparative clinical and prognostic significance of integrin αvβ3, αvβ5, and αvβ6 in osteosarcoma
Source: Front Oncol. 2026 Aug 4;16:1888679. doi: 10.3389/fonc.2026.1888679 (PMC13481227; doi:10.3389/fonc.2026.1888679)
Supplement: Supplementary file 1 [file Table1.docx]

**Supplementary Figure S1. Correlation analyses among αvβ3, αvβ5, and αvβ6 H-scores in osteosarcoma.** (A) Correlation between αvβ3 and αvβ6. (B) Correlation between αvβ3 and αvβ5. (C) Correlation between αvβ5 andαvβ6. Spearman correlation coefficients (ρ) and corresponding p values are indicated in each panel.

**Supplementary Figure S2. Kaplan–Meier survival analyses of αvβ5 and αvβ6 expression in osteosarcoma.** (A,B) EFS and MFS according to low and high αvβ5 expression groups. (C,D) Event-free survival (EFS) and metastasis-free survival (MFS) according to low and high αvβ6 expression groups. Patients were divided into low- and high-expression groups based on the median H-score of each marker.

**Supplementary Figure S3. Exploratory combined stratification analyses of the three-integrin panel in osteosarcoma.** (A,B) Kaplan–Meier curves for EFS and MFS according to the eight-group classification based on all possible low/high combinations of αvβ3, αvβ5, and αvβ6 expression. (C,D) Kaplan–Meier curves according to the number of highly expressed integrins (0–1 high vs. 2–3 high). (E,F) Kaplan–Meier curves according to the six-group joint classification model based on the combined expression patterns of the three markers.
